# Secreted Protein Acidic and Rich in Cysteine (SPARC) Suppresses Angiogenesis by Down-Regulating the Expression of VEGF and MMP-7 in Gastric Cancer

**DOI:** 10.1371/journal.pone.0044618

**Published:** 2012-09-05

**Authors:** Jun-Ling Zhang, Guo-Wei Chen, Yu-Cun Liu, Peng-Yuan Wang, Xin Wang, Yuan-Lian Wan, Jing Zhu, Hong-Qiao Gao, Jie Yin, Wei Wang, Mao-Lin Tian

**Affiliations:** Department of General Surgery, Peking University First Hospital, Beijing, People’s Republic of China; UAE University, United Arab Emirates

## Abstract

**Background:**

Secreted protein acidic and rich in cysteine (SPARC) is a glycoprotein that functions to inhibit angiogenesis, proliferation, and invasion in different types of cancer. The ability of SPARC to modulate neovascularisation is believed to be mediated in part by its ability to modulate the expression of vascular endothelial growth factor (VEGF) and matrix metalloproteinases (MMPs). In this study, we aimed to determine the effect of SPARC expression in gastric cancer cells on proliferation and angiogenesis *in vitro* and *in vivo*.

**Method:**

We evaluated expression of SPARC in seven human gastric cancer cell lines. Then we established a stably transfected SPARC overexpressed cell line (BGC-SP) and a stably transfected SPARC knock-down cell line (HGC-sh). The effect of SPARC overexpression and SPARC silencing was studied by examining capillary formation of HUVECs *in vitro* and a dorsal skin-fold chamber model *in vivo*. Quantitative real-time PCR and western blotting were performed to detect if the expressions of VEGF and MMP-7 were modulated by SPARC expression. To further determine the effect of SPARC expression on angiogenesis *in vivo*, xenograft models were established and microvessel density (MVD) of different clones were detected by immunohistochemistry.

**Results:**

Endogenous SPARC overexpression inhibited the expression of VEGF and MMP-7, as well as the angiogenesis induced by BGC-SP cells. Correspondingly, SPARC silencing increased the expression of VEGF and MMP-7, as well as the angiogenesis induced by HGC-sh cells. Elevated angiogenesis induced by SPARC silencing in HGC-sh cells was decreased when VEGF was neutralised by antibodies, and MMP-7 was knocked down *in vitro*.

**Conclusion:**

SPARC suppresses angiogenesis of gastric cancer by down-regulating the expression of VEGF and MMP-7.

## Introduction

Among cancer-related deaths, gastric cancer ranks second worldwide after lung cancer; almost two-thirds of the cases occur in developing countries, including 42% from China [1]. Angiogenesis is a critical process in gastric cancer; therefore, regulatory and signalling molecules that modulate angiogenesis are becoming the focus of present research. Angiogenesis is not an active process by itself; it is controlled by some angiogenic factors and some inhibitors of angiogenesis.

Secreted protein acidic and rich in cysteine (SPARC), also known as Osteonectin or BM-40, is a multi-faceted secreted glycoprotein which is expressed by many different types of cells and is associated with bone formation, fibrosis and tissue repair.

Recent studies show that SPARC modulates proliferation, apoptosis, invasion and angiogenesis in different types of cancer cells, however, the role of SPARC in tumourigenesis is complicated and seems to be cell-type specific owing to its diverse functions in a given micro-environment [2]. SPARC functions as a tumour suppressor in breast, neuroblastoma, pancreatic, ovarian and lung cancers [3]. Ovarian cancer in SPARC-null mice grew significantly larger than that in wild-type animals with augmented levels of vascular endothelial growth factor (VEGF) and matrix metalloproteinases (MMPs) [4]. By suppressing tumour vascularity through suppression of VEGF expression and secretion, SPARC inhibited glioma growth [5]. SPARC binds to VEGF, thus inhibiting VEGFR phosphorylation, mitogen-activated protein kinases (MAPK) activation and VEGF-induced DNA synthesis [6]. However, the role of SPARC in angiogenesis is also cell-type specific, which alters signal transduction events in response to unique cellular milieus [7].

VEGF stimulates angiogenesis, and is the most important signal protein produced by cells [8]. MMPs play important roles in tumour development, not only in degrading the extracellular matrix but also in regulating angiogenesis. MMP-7, which is the smallest molecular weight of all MMP family members, has been shown to accelerate the proliferation of human umbilical vein endothelial cells (HUVECs) in a dose-dependent manner *in vitro* [9].

The primary function of SPARC in angiogenesis of gastric cancer cell lines remains unclear. Therefore, in this study, we hypothesized that SPARC might modulate proliferation and angiogenesis by regulating VEGF and MMP-7 expressions in gastric cancer cells. To test these hypotheses, we tested expression of SPARC in seven gastric cancer cell lines. Then, to assess the effect of altered SPARC on gastric cancer cells, we established a BGC-SP clone which overexpressed SPARC and a HGC-sh clone in which the endogenous SPARC was knocked down.

## Results

### Expression of SPARC in Cultured Gastric Cancer Cells

**Figure 1:**
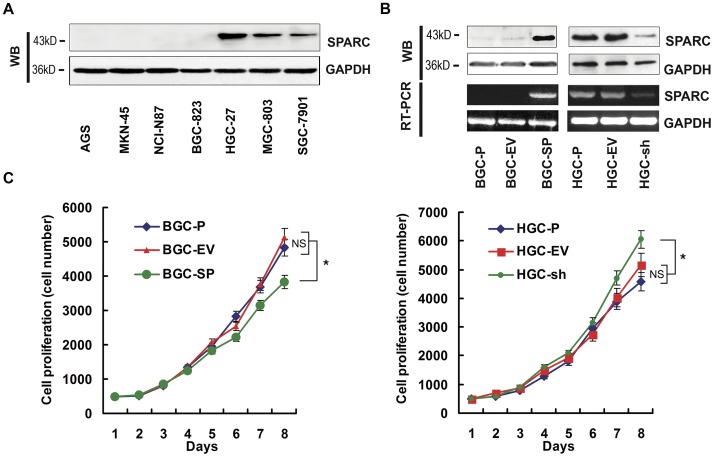
SPARC expression in gastric cancer cell lines, and the effects of SPARC expression alteration on cell proliferation. (**A**) SPARC protein expression in 7 types of gastric cancer cell lines. (**B**) SPARC protein (upper panel) and mRNA (lower panel) expression in parental cells (BGC-P and HGC-P), empty vector transfected clones (BGC-EV and HGC-EV), SPARC cDNA–expressing clone (BGC-SP) and SPARC shRNA-expressing clone (HGC-sh). GAPDH served as loading control. (**C**) Cell proliferation was determined by MTT assay. BGC-P and HGC-P cells and transfected clones (500 cells) were cultured in 96-well plates and incubated for 8 days, and cell proliferation was assayed by MTT method. Results are shown as means (±s.d.) of quadruplicate determinations from six separate experiments.

We evaluated expression of SPARC in several human gastric cancer cell lines. Western blotting showed that the SPARC was undectable in AGS, MKN45, NCI-N87 and BGC-823 cell lines, however SGC-7901 cell line expressed low level of SPARC and HGC-27, MGC-803 cell lines expressed high level of SPARC (Figure 1A).

### Overexpression and Inhibition of Endogenous SPARC in Gastric Cancer Cell Lines

Western blotting showed that the 43 kDa band corresponding to the SPARC protein was significantly increased in BGC-SP (BGC cells expressing SPARC cDNA) cells compared with parental (BGC-P) and control cells transfected with the empty vector (BGC-EV) (P<0.05); the SPARC was inhibited by nearly two-thirds in the HGC-sh cells (HGC cells expressing SPARC-shRNA) compared with HGC-P and HGC-EV cells (P<0.05, Figure 1B). RT-PCR indicated that SPARC mRNA expression in BGC-SP cells was increased as compared with BGC-P and BGC-EV cells (P<0.05); the SPARC mRNA expression in HGC-sh decreased by nearly 80% as compared with HGC-P and HGC-EV cells (P<0.05, Figure 1B).

### SPARC Overexpression Decreases Proliferation of Gastric Cancer Cell Lines

To determine whether altered SPARC expression affected the proliferation of gastric cancer cell lines, the growth of transfected cells were compared with those of parental and empty vector controls. The data showed that the growth of BGC-SP cells was inhibited in comparison with BGC-P and BGC-EV cells after 8 days of culture (P<0.05); the growth of HGC-sh cells was increased slightly compared with HGC-P and HGC-EV cells (P<0.05, Figure 1C).

### SPARC Overexpression in Gastric Cancer Cell Lines Decreases Angiogenesis *in vitro* and *in vivo*

**Figure 2:**
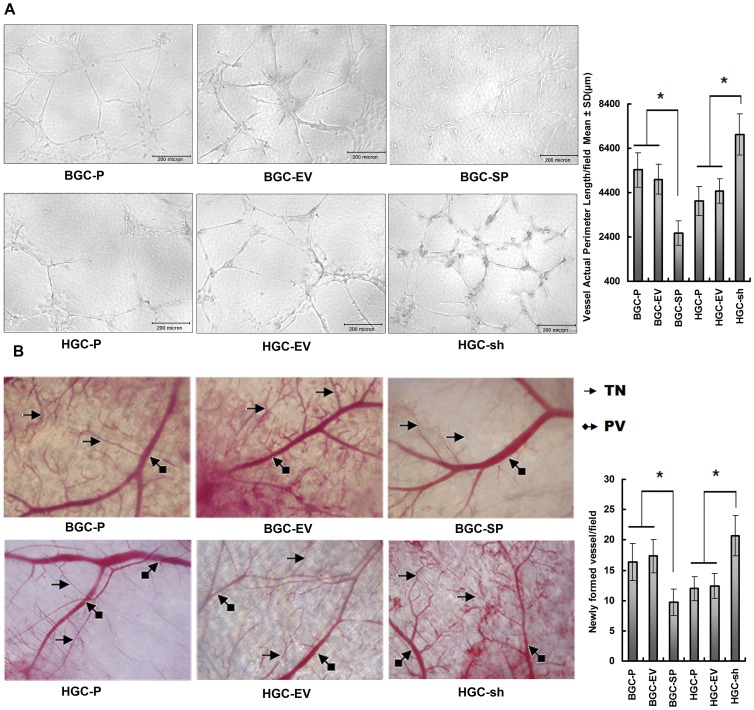
Overexpressed SPARC in gastric cancer cell lines decreases angiogenesis *in vitro* and *in vivo*. (**A**) *In vitro* angiogenesis: HUVECs were seeded in Matrigel-coated 96-well plates incubated with supernatant harvested from BGC-P and HGC-P cells or transfected clones. The cells were allowed to culture for 36 h on Matrigel in conditional media. The effects of conditioned media on the capillary formed by HUVECs were analysed, and the capillary length was measured. Capillary length data shown are means (±s.d.) of quadruplicate determinations from three separate experiments. *P<0.05, significant difference from control cells. (**B**) *In vivo* angiogenesis: BGC-P, BGC-EV, BGC–SP, HGC-P, HGC –EV, or HGC–sh (1×10^6^) were implanted into diffusion chambers and surgically placed underneath the dorsal skin of athymic nude mice. PV, pre-existing vasculature; TN, tumour-induced vasculature. Newly formed vessels were quantified and represented as per field. Columns are means (±s.d.) of quadruplicate fields from three separate experiments. *P<0.05, significant difference from control cells.

To understand the effect of altered SPARC expression on angiogenesis in gastric cancer cell lines, HUVECs were incubated in conditioned media. The BGC-SP supernatant induced HUVECs to differentiate into capillary-like structures within 36 h (2564.5±553.1 µm, P<0.05) to a lesser extent than the supernatant from BGC-EV cells (5002.4±665.7 µm) and BGC-P cells (5417.3±784.25 µm, Figure 2A). The HGC-sh supernatant induced HUVECs to differentiate into capillary-like structures within 36 h (7024.9±923.1 µm, P<0.05) to a stronger extent than the supernatant from HGC-EV cells (4456.2±554.2 µm) and HGC-P cells (4023.4±665.2 µm, Figure 2A). Quantification of the average tube length indicated that the tube length of HUVECs in conditioned media from BGC-SP was decreased by approximately 52.7% as compared with control cells; tube length of HUVECs in conditioned media from HGC-sh clones was increased 74.6% as compared with control cells (Figure 2A).

The dorsal window model showed that BGC-SP cells had a 40.4% decrease in tumour-induced microvessels as compared with control cells (P<0.05). HGC-sh cells in the dorsal skin-fold chamber resulted in a 73.2% increase in tumour-induced microvessels, with a greater number of tiny bleeding spots as compared with control cells (P<0.05 Figure 2B). These results clearly showed that SPARC overexpression in gastric cancer inhibited angiogenesis *in vitro* and *in vivo*.

### The MAPKs Signalling Pathway and Expression of VEGF and MMP-7 are Inhibited by SPARC Overexpression

**Figure 3:**
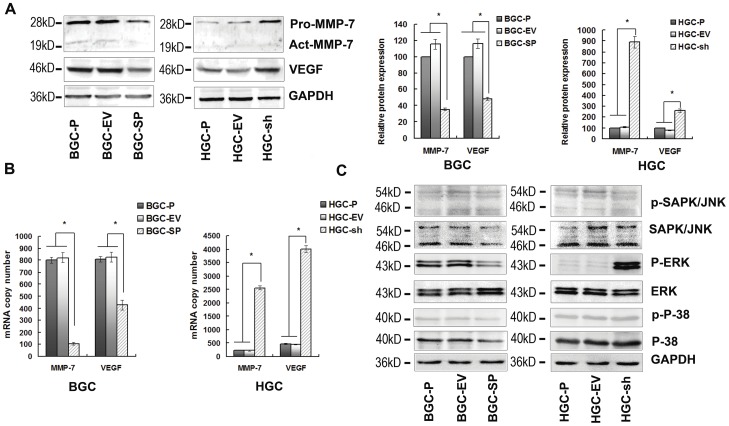
The MAPKs signalling pathway and expression of VEGF and MMP-7 are decreased by SPARC overexpression. (**A**) Cell lysates were used to perform western blotting analysis for VEGF and MMP-7 expression. GAPDH served as loading control. The expressions of VEGF and MMP-7 were declined at protein level in BGC-SP cells. The expressions of VEGF and MMP-7 were increased at protein level in HGC-sh cells. Columns are means (±s.d.) of triplicate experiments; *P<0.05, significant difference from control cells. (**B**) Real-time PCR was performed for assessment of VEGF and MMP-7 mRNA transcript levels. GAPDH served as a loading calibration. Columns are means (±s.d.) of triplicate experiments; *P<0.05, significant difference from control cells. (**C**) Cell lysates were used to perform western blotting analysis for molecules in MAPK signalling pathway. The activation of ERK1/2 was inhibited in BGC-SP cells compared with control cells, however, ERK1/2 was activated in HGC-sh cells compared with control cells.

To determine the effect of SPARC overexpression on MMP-7 and VEGF, quantitative real-time PCR and western blotting assays were performed. The results showed that MMP-7 and VEGF expression was negatively regulated by SPARC expression. In BGC-SP cells, levels of MMP-7 mRNA, MMP-7 protein, VEGF mRNA, and VEGF protein were inhibited by 87.2%, 68.9%, 48.4%, and 58.6%, respectively, as compared with empty vector transfected cells. In HGC-sh cells, the MMP-7 mRNA level increased 11.6-fold, the MMP-7 protein level increased 8.1-fold, the VEGF mRNA level increased 8.8-fold, and the VEGF protein level increased 3.2-fold as compared with empty vector cells (Figure 3A, B). To determine whether the MAPK signalling pathway was regulated by SPARC, SAPK/JNK, ERK1/2 and p-38 levels were assessed by western blotting. The results showed that levels of p-ERK1/2 were significantly decreased in BGC-SP cells and elevated in HGC-sh cells in comparison with their control cells (Figure 3C).

### Knock-down of SPARC Expression in HGC-27 Cells Promotes Angiogenesis via Up-regulated VEGF and MMP-7 Expression

**Figure 4:**
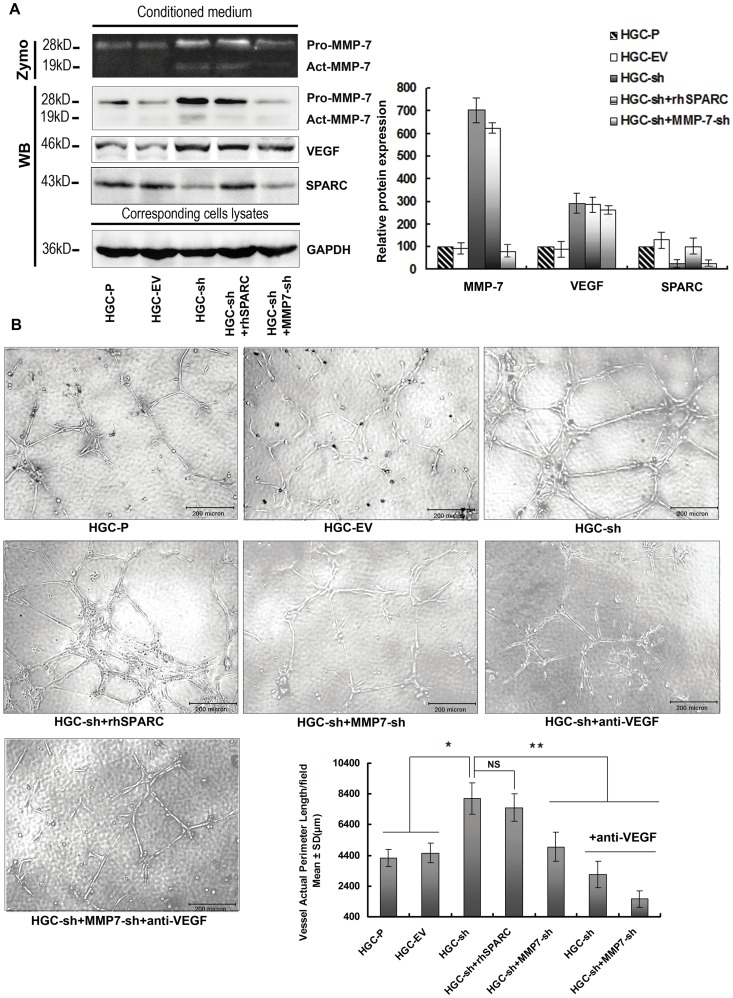
Knock-down of SPARC expression in HGC-27 cells promotes angiogenesis via up-regulated VEGF and MMP-7 expression. (**A**) Conditioned media from HGC-P, HGC-EV, HGC-sh with or without rhSPARC (0.3 µg/ml) and HGC-sh+MMP7-sh cells were concentrated under the same conditions. β-casein zymography was conducted for MMP-7 activity. Western blotting analysis was performed for MMP-7, SPARC and VEGF protein levels in conditioned media. The cells were collected and lysates probed with GAPDH antibody to calibrate total amount of the respective proteins. Columns are means (±s.d.) of triplicate experiments. (**B**) *In vitro* angiogenesis: To confirm that SPARC expression-mediated anti-angiogenic effects are due to altered MMP-7 and VEGF expression rather than to the expression of SPARC itself, harvested supernatant from HGC-sh cells was added to 0.3 µg/ml rhSPARC. Supernatants from both of HGC-sh and HGC-sh+MMP7-sh cells with neutralising antibody to VEGF were also used in co-culture assay (anti-VEGF =  neutralising antibody to VEGF). HUVECs were seeded in Matrigel-coated 96-well plates incubated with conditioned media. The effects of conditioned media on the pre-formed tubes of HUVECs were analysed, and the tube length was measured. Tube length data shown are the means (±s.d.) of quadruplicate determinations from three separate experiments. *P<0.05, significant difference from HGC-P cells, **P<0.05, significant difference from HGC-sh cells.

To confirm that the pro-angiogenic effects seen with decreased SPARC expression are due to increased MMP-7 and VEGF expression and not to levels of SPARC itself, HUVECs were incubated in conditioned media harvested from HGC-sh cells with exogenous added recombinant human SPARC (rhSPARC, 0.3 µg/ml). Our data indicated that adding exogenous SPARC did not inhibit capillary-like structures of HUVECs compared with HGC-sh (Figure 4), unlike the anti-angiogenic response seen with endogenous expression of SPARC in BGC823 cells (Figure 2).

To further characterise the role of VEGF and MMP-7 in SPARC-mediated angiogenesis modulation, MMP-7-shRNA and 1 µg/ml neutralising VEGF antibody (Chemicon, Temacula, CA, USA) were used for HGC-sh clones to antagonise the functions of MMP-7 and VEGF.

We examined the ability of MMP-7 expression in HGC-sh cells to modulate angiogenesis *in vitro* by stably transfecting MMP-7-shRNA into HGC-sh cells. Figure 4A indicates that the expression of MMP-7 in HGC-sh+MMP7-sh cells was down-regulated by stably expressing MMP-7-sh-RNA to a level comparable with that of HGC-P and HGC-EV cells. To elucidate the role of MMP-7 in knock-down SPARC-mediated promotion of tumour cell-induced angiogenesis, we performed capillary formation analysis with conditioned media of HGC-sh cells and HGC-sh+MMP7-sh cells. As shown in Figure 4B, results indicate that decreased MMP-7 expression in HGC-sh+MMP7-sh cells led to a significantly decreased capillary formation by HUVECs *in vitro* (HGC-sh+MMP7-sh *vs* HGC-sh, P<0.05).

To determine the function of elevated VEGF expression induced by SPARC silencing, VEGF in the conditioned media of HGC-sh and HGC-sh+MMP7-sh cells was neutralised by VEGF antibody (1 µg/ml). Results showed that capillary formation of HUVECs was decreased significantly in the HGC-sh supernatant containing the VEGF neutralising antibody as compared with supernatant from HGC-sh cells alone (HGC-sh + anti-VEGF *vs* HGC-sh, P<0.05 Figure 4B). Capillary formation of HUVECs was almost completely inhibited when cultured in conditioned media of HGC-sh+MMP7-sh cells plus added VEGF neutralising antibody (*vs* HGC-sh, P<0.05 Figure 4B).

Serum-free conditioned media harvested from HGC-P, HGC-EV, HGC-sh with or without rhSPARC (0.3 µg/ml) and HGC-sh+MMP7-sh cells were concentrated by ultrafiltration tube (Millipore, Bedford, MA, USA) under the same conditions. Western blotting showed that the concentration of SPARC in HGC-sh cells with 0.3 µg/ml rhSPARC inmedium was equal to that of the HGC-P supernatant (Figure 4A).

### Overexpression of SPARC in Gastric Cancer Cells Inhibits Tumourigenicity in Nude Mice

**Figure 5:**
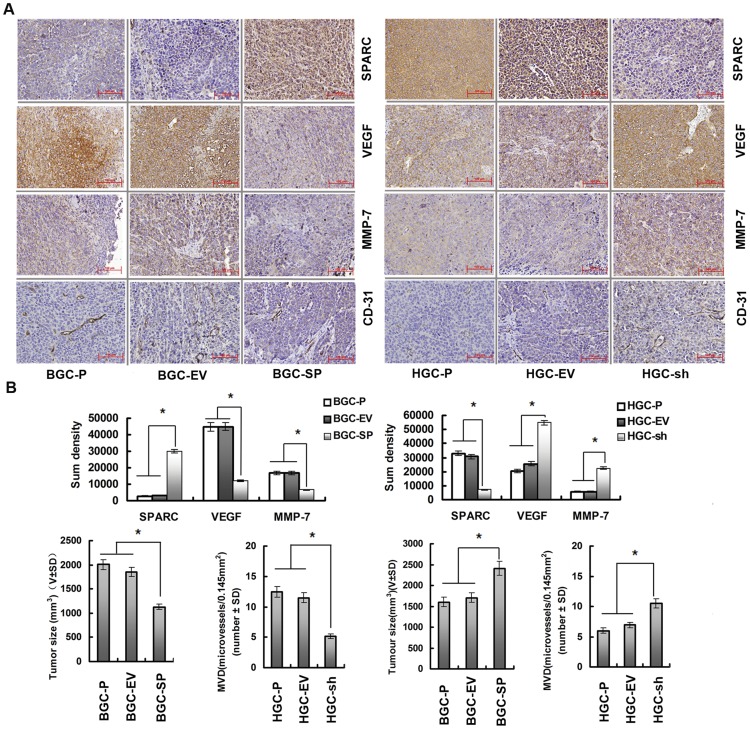
Overexpression of SPARC in gastric cancer cells inhibits tumour development and vascularisation in nude mice. (**A**) Paraffin-embedded sections of xenografted tumours were used for immunohistochemical analysis of SPARC, MMP-7, VEGF, and CD-31. (**B**) Sections were stained with a monoclonal antibody against human SPARC, VEGF, MMP-7. Sum densities were calculated and analyzed by IPP 6.0. Columns are means (±s.d.) of quadruplicate determinations from six mice in each group. *P<0.05, significant difference from control cells. (**C**) MVD in tumour tissues was quantified by counting CD31-positive areas in each microscopic field of view. Columns are means (±s.d.) of quadruplicate determinations from six mice in each group. *P<0.05, significant difference from control cells. The tumour volume was calculated (volume  =  width^2^× length× 0.52). The volume of tumours at the 50th day is indicated by the mean values (±s.d.) of six mice in each group, *P<0.05, significant difference from control cells.

To assess the therapeutic efficacy of SPARC expression, BGC-P, BGC-EV, BGC-SP cells or HGC-P, HGC-EV, HGC-sh cells were injected subcutaneously into nude mice. There was no significant difference in size between BGC-P (n = 6; mean tumour volume = 2004±63 mm^3^), BGC-EV (n = 6; mean tumour volume = 1856±69 mm^3^) xenografts. A significant decrease (39.1%) in mean tumour volume was found in animals implanted with BGC-SP xenografts (n = 6; mean tumour volume = 1130±55 mm^3^) as compared with animals implanted with BGC-EV xenografts (P<0.05, Figure 5). There was no significant difference in size between HGC-P (n = 6; mean tumour volume = 1605±63 mm^3^), HGC-EV (n = 6; mean tumour volume = 1708±82 mm^3^) xenografts. A significant increase (50.3%) in mean tumour volume was found in animals implanted with HGC-sh xenografts (n = 6; mean tumour volume = 2412±75 mm^3^) as compared with animals implanted with HGC-EV xenografts (P<0.05, Figure 5).

To assess SPARC, VEGF, MMP-7 expressions *in vivo*, xenograft sections were stained with a monoclonal antibody against human SPARC, VEGF or MMP-7. Figure 5A indicates that BGC-SP tumours express more SPARC than BGC-P, BGC-EV tumours, while concomitantly VEGF, MMP-7 expressions are decreased (P<0.05, Figure 5A). Sections from HGC-sh tumours express less SPARC than HGC-P, HGC-EV tumours while concomitantly VEGF, MMP-7 expressions are increased (P<0.05, Figure 5A). CD31 is used primarily to demonstrate the presence of vascular endothelial cells in histological tissue sections, which can help to evaluate the degree of tumour angiogenesis. To assess whether altered SPARC expression mediated the microvessel density (MVD), we analysed angiogenesis in xenografts by histological analysis of CD-31. There was no significant difference in MVD between BGC-P (12.5±2.3 microvessels/0.145 mm^2^), BGC-EV (11.5±3.4 microvessels/0.145 mm^2^) xenografts. MVD was decreased 54.8% in BGC-SP( 5.2±2.1 microvessels per/mm^2^) tumours compared with BGC-EV tumours (P<0.05, Figure 5). There was no significant difference in MVD between HGC-P (6.4±2.1 microvessels/0.145 mm^2^), HGC-EV (6.9±1.8 microvessels/0.145 mm^2^) xenografts. MVD was elevated 51.7% in HGC-sh (10.5±1.5 microvessels/0.145 mm^2^) tumours compared with HGC-EV tumours (P<0.05, Figure 5).

## Discussion

SPARC is a tissue-specific protein that affects multiple cell processes including proliferation, invasion, and angiogenesis variably in different types of tissues. For example, previous studies demonstrated that SPARC promoted the invasion while concomitantly inhibiting the growth of tumors [5], [10]. In medulloblastoma, the overexpression of SPARC can inhibit the angiogenesis in tumour by lowering the expression and secretion of VEGF and MMP-9 [11]. In melanoma, however, the expression of SPARC was positively correlated with angiogenesis [12]. The function of SPARC in gastric cancer cells remains unclear.

In order to investigate the role of SPARC in gastric cancer, we first tested the expression of SPARC in seven cell lines of gastric cancer. Most cell lines did not express, or only expressed low level of SPARC. To determine the role of SPARC in the growth and angiogenesis of gastric cancer, we established the BGC-SP clone which was stably transfected with a SPARC cDNA vector, and the HGC-sh clone which was stably transfected with a shRNA vector targeting SPARC mRNA. SPARC expression increased significantly in BGC-SP clone and decreased in HGC-sh clone in comparison with their respective control clones, as determined by western blotting and RT-PCR analyses. Cell proliferation rate was lower in BGC-SP clone, and was higher in HGC-sh clone than in their respective control clones by MTT method. We also found that overexpression of SPARC inhibited tumour cell-induced capillary formation of HUVECs *in vitro* and angiogenesis in dorsal window assay *in vivo*. On the other hand, down-regulation of SPARC by mRNA interference promoted capillary formation *in vitro* and angiogenesis *in vivo*.

Blood vessels are essential to deliver nutrients to tissues. Therefore, neovascularisation is indispensable to the development of solid tumour. Previous studies have demonstrated that SPARC plays a role in angiogenesis [7]. Our results showed that overexpression of SPARC inhibited angiogenesis *in vitro* and *in vivo* in association with the decrease of MMP-7, VEGF and phosphorylated ERK1/2, while down-regulation of SPARC promoted angiogenesis *in vitro* and *in vivo* in association with the increase of MMP-7, VEGF and phosphorylated ERK1/2.

We further implemented studies to investigate the role of VEGF and MMP-7 in SPARC-mediated angiogenesis modulation. When recombinant human SPARC protein was added to conditioned medium from HGC-sh clone to restore SPARC concentration, this conditioned medium did not change the capillary formation of HUVECs by *in vitro* assay compared to the capillary formation of HUVECs incubated in the condition medium without exogenous rhSPARC. We then used MMP-7-shRNA to down-regulate MMP-7 expression in HGC-sh clone, and/or anti-VEGF antibody to neutralize VEGF in conditioned medium from HGC-sh clone. Capillary formation of HUVECs was inhibited significantly when they incubated in the conditioned media with lower MMP-7 and/ or blocked VEGF. These experiments suggest that SPARC down-regulation alone is insufficient for the induction of neovascularisation, and other factors must be involved in this process.

VEGF plays a key role in angiogenesis, and is necessary for the survival of endothelial cell [8]. In glioma, SPARC inhibited tumour growth by altering its micro-environment and suppressing its angiogenesis through the inhibition of VEGF expression and secretion [5]. There may be a negative relationship between SPARC and VEGF expressions, i.e., the more SPARC, the less VEGF or *vice versa* [13], [14].

MMP-7 is capable of degrading basement membrane or connective tissue around the vessels. It also stimulates DNA synthesis in cultured vascular endothelial cells, and induces angiogenesis at the site where colon cancer cells were implanted in a mouse model [15]. VEGF and other angiogenic factors function mainly through MAPK signalling pathways, which are believed to be important transduction pathways involved in the neovascularisation processes in tumours [8]. Our recent study showed that MMP-7 expression was modulated via the activation of MAPK signalling pathways [16]. Several studies also demonstrated that SPARC negatively modulated the activation of MAPK pathways [17]. Consequently, SPARC expression may alter the angiogenic balance in tumours by down-regulating a series of neovascularisation promoting factors.

To investigate the function of SPARC in the regulation of gastric cancer growth *in vivo*, BGC-SP and HGC-sh cell clones were compared with their control clones for their ability to form tumours in a subcutaneous model. SPARC overexpression significantly reduced the size of xenografted tumour with reduced MVD, down-regulation of SPARC by RNA interference promoted the growth of xenografted tumour with increased MVD. Therefore, in gastric cancer xenografts, SPARC expression is negatively correlated with angiogenesis. Previous studies indicated that SPARC contributed to the regulation of tumour formation, although its role seemed to be cell-type specific. In hepatocellular cancer cell-line xenografts, SPARC overexpression significantly delayed tumour formation, reduced tumour size, and decreased MVD in comparison with control xenografts [18]. In colon cancer tissues, SPARC expression was negatively correlated with VEGF expression and MVD [19]. In medulloblastoma cells, SPARC overexpression inhibited angiogenesis leading to the decrease of tumour growth [11]. In human microvascular endothelial cells, SPARC inhibited DNA synthesis *in vitro* [6]. In neuroblastoma xenografts, SPARC peptides inhibited angiogenesis and tumour growth *in vivo* [20]. These results confirmed SPARC as an inhibitor of tumour angiogenesis *in vivo*.

SPARC expresses in normal gastric epithelial cells, gastric cancer cells, and the stromal cells surrounding gastric cancer at a lower level [21]. An immunohistochemistry study showed that SPARC mainly expressed in stromal cells surrounding the tumour [22]. These discrepancies cannot be fully explained. SPARC expression may depend on histological type of the tumour, or *vice versa*. Recent immunohistochemistry study found that SPARC expression was negatively correlated with the expression of VEGF and MVD in gastric cancer tissues, and SPARC expression decreased in gastric cancer with higher grade of malignancy [23].

In summary, the growth inhibition of gastric cancer by SPARC seems to be mediated through its suppression effects on MMP-7 and VEGF expressions, which may in turn inhibit microvessel infiltration into tumours. We conclude that down-regulation of SPARC may associate with the progress of gastric cancer, and the exploration aimed to regulate SPARC expression may become a meaningful approach to improve gastric cancer treatment.

## Materials and Methods

### Antibodies and Reagents

Antibodies against SPARC (Santa Cruz Biotechnology, Santa Cruz, CA, USA), (p-)SAPK/JNK, (p-)ERK1/2, (p-)p-38, MMP-7 (Cell signaling technology, Danvers, MA,USA), VEGF, and CD31 (ABcam, Cambridge, MA , USA) were used for western blotting and immunohistochemistry. The rhSPARC was supplied by R&D (Minneapolis, MN, USA). Reverse transcription-PCR kit was supplied by Promega (Madison, WI, USA). MMP-7-shRNA (KH00809P, SuperArray Bioscience Corp. Frederick, MD, USA) was used for the down-regulation of MMP-7 in the cell clones. β-Casein (C-6905, Sigma-Aldrich Corporation, Natick, MA, USA) was used in β-casein zymography. All other reagents were of analytical grade or better.

### Cell Culture

Human gastric cancer cell lines AGS, MKN-45, NCI-N87, BGC823, MGC803, HGC27, SGC7901 were obtained from the Cancer Institute of the Chinese Academy of Medical Science. All cells were grown in RPMI 1640 medium supplemented with 10% fetal bovine serum (FBS). BGC-EV (transfected with empty vector), BGC-SP (overexpressing SPARC cDNA), HGC-EV (expressing empty vector) and HGC-sh (expressing SPARC shRNA) were grown in complete RPMI 1640 with G418 (50 µg/ml). All cells were maintained in monolayer cultures at 37°C in humidified air with 5% CO_2_.

### Establishment of BGC-SP, HGC-sh Clones and HGC-sh-MMP7-sh Clones

Approximately 150,000 BGC-823 cells were plated per well in a six-well plate in RPMI 1640 with 10% FBS and allowed to attach overnight. Equimolar amounts of pcDNA3.1 with full-length SPARC cDNA vector or the empty vector (Invitrogen, San Diego, CA, USA) were incubated with Lipofectamine-2000 Transfection Reagent (Invitrogen, San Diego, CA, USA). Validated SureSilencing human SPARC shRNA and empty control vector were obtained from SuperArray Bioscience Corp. (Frederick, MD, USA). HGC-27 cells were transfected as described previously [24]. Briefly, cells were transfected in a stable manner using lipofectamine. Transfected cells were selected with G418 (100 µg/ml for BGC-SP and HGC-sh clones) for 14 days before the isolation of individual clones. HGC-sh-MMP7-sh variants were established as described above, HGC-sh clone cells were transfected with MMP-7-shRNA (KH00809P, SuperArray Bioscience Corp. Frederick, MD, USA) using lipofectamine. Then, transfected cells were selected by puromycin (1 µg/ml) for 10 days.

### Cell Proliferation Assay

Cell proliferation was determined by a 3-(4,5-Dimethylthiazol-2-yl)-2,5-diphenyltetrazolium bromide (MTT) assay as described previously [24]. Briefly, 500 cells were cultured per well in 96-well plates and incubated for 8 days, and then, MTT (R&D, Minneapolis, MN, USA) was added to the cells. Absorbance values at 550 nm were measured with a microplate reader. The results were shown as mean absorbance at 550 nm and the means (±s.d.) of quadruplicate determinations from six separate experiments.

### Western Blotting Analysis

Total cell lysates were prepared and analysed by western blotting as previously described [24]. Briefly, antibodies to SPARC, MMP-7, and VEGF (1∶1000 dilution) were used to detect SPARC, MMP-7, and VEGF, respectively, while antibodies to (p-)SAPK/JNK, (p-)ERK1/2 and (p-)p-38 (1∶800 dilution) were used to detect activation of the MAPK signalling pathway. Bound antibodies were visualised using ECL (Promega, Madison, WI, USA) on a Kodak Image Station 4000 mm Pro System (Kodak, Rochester, NY, USA). The density of the bands was quantified by densitometric analysis using the Image Tool (version 3.0) system.

### RT-PCR and Quantitative Real-time PCR

Total RNA was isolated from stably transfected tumour cells using Trizol reagent (Invitrogen, San Diego, CA, USA) and treated for 45 min at 37°C with RQ1 DNase (Promega, Madison, WI, USA). RNA was reverse transcribed using AMV Reverse Transcriptase (A3500, Promega, Madison, WI, USA). Quantitative real-time PCR was performed in an ABI Prism7300 Sequence Detection System (Applied Biosystems, Beverly, MA, USA) using a GoTaq qPCR Master Mix A6001 kit (Promega, Madison, WI, USA). Primers used for quantitative real-time PCR were as follows: MMP-7, 5′-GGAGATGCTCACTTCGATGA-3′ (sense) and 5′-ATACCCAAAGAATGGCCAAG-3′ (antisense); and VEGF, 5′-AGGAGGAGGGCAGAATCATCA-3′ (sense) and 5′-CTCGATTGGATGGCAGTAGCT-3′ (antisense). Primers used for PCR were as follows: SPARC, 5′-CTCGAGATGAGGGCCTGGATCTTC-3′ (sense) and 5′-GGATCCCGGATCACAAGATCCTTGTCG-3′ (antisense); and glyceraldehyde-3-phosphate dehydrogenase (GAPDH), 5′-GGAGTCCACTGGCGTCTTC-3′ (sense) and 5′-GCTGATGATCTTGAGGCTGTTG-3′ (antisense).

### β-casein Zymography

The functional activity of MMP-7 was evaluated by β-casein zymography on 10% polyacrylamide gels embedded with 1 mg/ml β-casein. Equal amounts of the serum-free conditioned media from cells grown for 24 hours were electrophoresed. After electrophoresis, the gels were washed in 2.5% Triton X-100 for one hour to remove SDS. The gels were then incubated for 18 hours at 37°C in 50 mM Tris/HCl containing 10 mM CaCl_2_ and 0.02% NaN_3_, stained with coomassie brilliant blue and then destained. Proteolytic activities of latent MMP-7 and activated MMP-7 were evidenced as bands with molecular masses of 28 and 19 kDa, respectively.

### Conditioned Media Collection for Experimentation

In total, 2×10^5^ cells of HGC-P, BGC-P or their corresponding stably transfected clones were seeded and incubated in complete RPMI 1640 in 6-well chamber slides and allowed to grow for 24 h. Subsequently, conditioned media were collected, labelled and stored at −80°C for future use.

### Endothelial Cell Capillary-like Tube Formation Assay

To examine the effect of SPARC on *in vitro* angiogenesis, a capillary formation assay was performed. In this assay, matrigel was pipetted into pre-chilled 96-well plates (75 µl matrigel per well) and polymerised for 30 min at 37°C. To determine if altered SPARC expression would regulate angiogenesis, HUVECs (5000 cells per well) were incubated in 100 µl of conditioned media harvested from different kinds of cells. After 36 h of incubation, tubular structures were photographed. Each assay condition was assessed in quadruplicate determinations from three separate experiments. Images were captured using a Cannon Power Shot A640 camera on an Olympus inverted microscope with a 100× magnification; the tube length was quantified using IPP (version 6.0, Media Cybernetics, Silver Spring, MD).

### Dorsal Skin-fold Chamber Model

The studies were performed in accordance with a protocol approved by the Animal Care and Use Committee of the Peking University (ethical application approval number No. J201155). The *in vivo* procedures were in compliance with the recommendations in the Guide for the Care and Use of Laboratory Animals of the National Institutes of Health. Athymic nude mice (6 weeks old, female, 26–28 g, n = 3 per group) were bred and maintained within a germ-free environment. The implantation technique has been described previously [11]. A dorsal air sac was made in the mouse by injecting 10 ml of air subcutaneously after the animal was anaesthetised completely. Diffusion chambers (Millipore, Bedford, MA, USA) were prepared by aligning 0.45-mm Millipore membranes on both sides of the rim of the ‘O’ ring with cement. BGC-P, BGC-EV and BGC-SP; HGC-P, HGC-EV or HGC-sh cells (1×10^6^) suspended in PBS were injected into the chamber. A 2 cm long incision was made horizontally along the edge of the dorsal air sac, and the chambers were placed underneath the skin. The mice were sacrificed 10 days later. The animals were carefully skinned around the implanted chambers. The skin folds covering the chambers were photographed under visible light. The number of blood vessels was counted.

### Xenograft Models and Immunohistochemistry

The athymic nude mice were randomised to different groups (n = 6 per group). Mice were inoculated subcutaneously in the lower rear flank with human BGC-P, BGC-EV, BGC-SP or HGC-EP, HGC-EV, HGC-sh cells (2×10^6^ cells per mouse). Tumour growth was monitored by palpation at the site of inoculation. The tumour size was measured every 5 days with calipers, and the tumour volume was calculated (volume  =  length × width^2^ × 0.52). Mice were monitored for 50 days, which was the termination point of the experiment. Tumours were excised and fixed in formalin at the optimal cutting temperature for further histological analysis.

Immunohistochemistry staining was performed on formalin-fixed and paraffin-embedded material. Four-micrometre sections were deparaffinised and subjected to antigen retrieval at 100°C for 20 min in 1 mmol/L EDTA buffer (pH 8.0) in a microwave-oven. Sections were incubated at 4°C overnight with SPARC, MMP-7, VEGF and CD31 antibodies (1∶100 dilution). SPARC, VEGF and MMP-7 densities were analyzed by IPP (version 6.0, Media Cybernetics, Silver Spring, MD). The density (sum) was calibrated using the method introduced by Xavier [25]. CD31 was used primarily to demonstrate the presence of vascular endothelial cells in histological tissue sections. MVD was calculated using quantification of CD31-positive microvessels per field of view. Quantification was assessed at MVD hotspots using at least 24 fields of view for each tumour at 400× magnification. Images were captured using an Olympus DP71 camera on Olympus BX51 microscopes and imaging systems.

### Statistical Analysis

All data were expressed as the mean±s.d. Statistical analysis was performed using one-way ANOVA followed by Dunnett’s multiple comparison or a Student’s t-test. P<0.05 was considered statistically significant. All tests were carried out with SPSS 13.0.
